# Methods in Nutrition & Gut Microbiome Research: An American Society for Nutrition Satellite Session [13 October 2022]

**DOI:** 10.3390/nu15112451

**Published:** 2023-05-24

**Authors:** Riley L. Hughes, Cara L. Frankenfeld, Daryl M. Gohl, Curtis Huttenhower, Scott A. Jackson, Doris Vandeputte, Emily Vogtmann, Sarah S. Comstock, Mary E. Kable

**Affiliations:** 1 Independent Researcher, Seattle, WA 98115, USA; rlh13@illinois.edu; 2 Public Health Program, University of Puget Sound, Tacoma, WA 98416, USA; cfrankenfeld@pugetsound.edu; 3 University of Minnesota Genomics Center, Minneapolis, MN 55455, USA; dmgohl@umn.edu; 4 Department of Genetics, Cell Biology, and Developmental Biology, University of Minnesota, Minneapolis, MN 55455, USA; 5 Department of Biostatistics and Immunology and Infectious Diseases, Harvard T.H. Chan School of Public Health, Boston, MA 02115, USA; chuttenh@hsph.harvard.edu; 6 Broad Institute of MIT and Harvard, Cambridge, MA 02142, USA; 7 Harvard Chan Microbiome in Public Health Center, Harvard T.H. Chan School of Public Health, Boston, MA 02115, USA; 8 Complex Microbial Systems Group, Biosystems and Biomaterials Division, National Institute of Standards and Technology, Gaithersburg, MD 20899, USA; scott.jackson@nist.gov; 9 Meinig School of Biomedical Engineering, Cornell University, Ithaca, NY 14850, USA; dvv5@cornell.edu; 10 Metabolic Epidemiology Branch, Division of Cancer Epidemiology & Genetics, National Cancer Institute, National Institutes of Health, Bethesda, MD 20892, USA; emily.vogtmann@nih.gov; 11 Department of Food Science and Human Nutrition, Michigan State University, East Lansing, MI 48824, USA; 12 USDA-ARS Western Human Nutrition Research Center, University of California—Davis, Davis, CA 95616, USA

**Keywords:** microbiota, methods, nutrition, sample collection, bioinformatics, sequencing

## Abstract

The microbial cells colonizing the human body form an ecosystem that is integral to the regulation and maintenance of human health. Elucidation of specific associations between the human microbiome and health outcomes is facilitating the development of microbiome-targeted recommendations and treatments (e.g., fecal microbiota transplant; pre-, pro-, and post-biotics) to help prevent and treat disease. However, the potential of such recommendations and treatments to improve human health has yet to be fully realized. Technological advances have led to the development and proliferation of a wide range of tools and methods to collect, store, sequence, and analyze microbiome samples. However, differences in methodology at each step in these analytic processes can lead to variability in results due to the unique biases and limitations of each component. This technical variability hampers the detection and validation of associations with small to medium effect sizes. Therefore, the American Society for Nutrition (ASN) Nutritional Microbiology Group Engaging Members (GEM), sponsored by the Institute for the Advancement of Food and Nutrition Sciences (IAFNS), hosted a satellite session on methods in nutrition and gut microbiome research to review currently available methods for microbiome research, best practices, as well as tools and standards to aid in comparability of methods and results. This manuscript summarizes the topics and research discussed at the session. Consideration of the guidelines and principles reviewed in this session will increase the accuracy, precision, and comparability of microbiome research and ultimately the understanding of the associations between the human microbiome and health.

## 1. Background

The human microbiome is a rapidly expanding area of research linked to health outcomes [1]. Hundreds to thousands of different microbial species colonize mucosal and skin environments of the human body, particularly in the human gut, and the sum of these host-associated microbes contains more genetic material than the human genome [2]. This complex microbial ecosystem is unique to each individual and can change over time in response to aging, medication or disease, changes in lifestyle factors such as diet, or changes in other environmental factors [2,3]. Critically, differences in gut microbiome composition or function have been linked to differences in human health outcomes and disease states, such as glycemic response, cancer, inflammatory bowel disease, and immune system complications ranging from allergy to rheumatoid arthritis [4,5].

Microbial community samples can be profiled using multiple types of molecular assays, including metagenomics (e.g., species, strains, genes), metatranscriptomics (e.g., gene expression), metaproteomics (e.g., proteins), and metametabolomics (e.g., metabolites) that provide information on both the composition and function of the microbiome. These data can then be normalized, integrated, and analyzed to detect associations with health outcomes. Validation studies in vivo (e.g., gnotobiotic mice) and in vitro (e.g., cell culture) can then be used both to support previous findings and understand mechanisms of action. Understanding these connections between the human microbiome and health outcomes will allow us to devise potential microbiome-targeted recommendations or treatments to help prevent or treat disease.

A prerequisite of understanding these connections is accurate, precise, and consistent measurement of microbiome communities as well as appropriate biostatistical methods to then relate those microbial measurements with phenotypes. Methods and technologies intended to achieve greater accuracy and precision of results are rapidly advancing and proliferating, resulting in a wide range of approaches that make it difficult to compare results across studies. Thus, the American Society for Nutrition (ASN) Nutritional Microbiology GEM, sponsored by the Institute for the Advancement of Food and Nutrition Sciences (IAFNS), brought together experts in the field of human microbiome studies to review the current state of knowledge related to the methods used for each step of microbiome analysis: collection, storage, sequencing, bioinformatic processing, and biostatistical analysis. The objectives of this satellite session were to review the pros and cons of different methods for each step, the context in which specific methods are appropriate, and the importance of synchronizing methods or implementing practices such as use of internal standards to increase inter-study comparability.

## 2. Microbiome Sample Collection and Storage Methods

Starting with the first steps of microbiome analysis: collection and storage, Dr. Emily Vogtmannn from the National Cancer Institute led with her talk entitled, “Methods for Fecal Sample Collection and Preservation: Considerations for Current and Future Studies”. The collection and preservation of fecal samples provide the starting point from which many other steps follow and therefore form one of the foundations of microbiome analysis.

Important criteria for a rigorous collection method are technical reproducibility, stability, and concordance [6]. Technical reproducibility is the similarity of different aliquots from the same fecal sample. Stability is the potential of the fecal sample once collected to remain constant or unaltered prior to freezing for a period of time. Fecal samples must often be collected at home and either brought or mailed into the lab before being frozen, which makes stability at ambient temperature an important aspect of a fecal collection method. Concordance refers to the comparison of each sample collection method to a “gold standard”. In the assessment of collection methods, Dr. Vogtmann and colleagues used immediately frozen samples as the putative gold standard to compare to samples held at room temperature for a period of time, although they acknowledge the limitations of this “gold standard” [6].

Dr. Vogtmann reviewed six different fecal collection methods: no additive, 70% ethanol, 95% ethanol (Sigma-Aldrich, St. Louis, MO, USA), RNAlater Stabilization Solution (Ambion, Austin, TX, USA), fecal occult blood test (FOBT) cards (Beckman Coulter, Brea, CA, USA), and fecal immunochemical test (FIT) tubes (Polymedco, Inc., Cortlandt Manor, NY, USA). Pros and cons of these different methods are shown in Table 1. Technical reproducibility among all methods was high, and therefore stability and concordance were the primary criteria compared between methods [7].

To assess stability at ambient temperature, intraclass correlation coefficients (ICC) were calculated, with zero being the least stable and one being the most stable, from 16S rRNA gene amplicon sequencing [7]. This assessment demonstrated high variability between methods, revealing the “no additive” samples to be the least stable (low ICC). Samples preserved in 70% ethanol also had relatively low stability. The other four methods: 95% ethanol, RNAlater, FOBT cards, and FIT tubes were all relatively similar and demonstrated high ICC values, indicating high stability of samples held at room temperature [7]. To extend these results, Dr. Vogtmann conducted shotgun metagenomic sequencing (MGX) and metabolomic measurements on a subset of these samples. When MGX was used, the samples with 95% ethanol had lower ICC values, while the other three previously stable methods (RNAlater, FOBT cards, and FIT tubes) had higher ICC values for stability [8]. In the metabolomic analysis, samples with RNAlater could not be used due to the high sodium sulfate content, which was incompatible with mass spectrometry-based metabolomics platforms [9]. Additionally, the FIT tubes showed low ICC stability values for metabolomic analyses, though samples collected using 95% ethanol or FOBT cards demonstrated high stability [9].

Concordance was assessed by comparing samples collected using these methods to the putative gold standard samples that were frozen immediately with no solution using both ICCs and Spearman correlation coefficients (SCC) [6,7,8,10,11]. The concordance of genus-level relative abundances differed by collection method and were typically low, but alpha- and beta-diversity metrics had relatively higher ICCs or SCCs when using 16S rRNA sequencing or MGX [6,7,8,10]. For metabolomic analysis, samples collected with 95% ethanol and the FOBT cards were the most accurate but samples with 95% ethanol had the greatest number of metabolites detected, indicating that this may be the best collection method for metabolomic analysis when immediately frozen fecal samples are not possible [9].

Additional factors such as shipping temperature and long-term storage stability were also discussed. Thus far, the comparison of collection methods has presumed storage of samples at room temperature. However, when shipping samples, temperature may vary by season. Thus, Dr. Vogtmann and colleagues investigated the impact of temperature (room temperature, 4 °C [winter shipping temperature], and 30 °C [summer shipping temperature]) on the stability of FIT tubes [12]. Though temperature differences did result in some differences in stability, all samples remained within acceptable ICC ranges [12]. In this study, genetic material collection cards kept at room temperature for 70 days also showed high stability [12].

For large cohort studies, when investigators are interested in prospectively evaluating microbiome associations with health outcomes, such as the development of cancer, using a nested design, samples must remain stable in the freezer for years. Thus, Dr. Vogtmann presented data from another study in which fecal samples were collected using no additive, 95% ethanol, RNAlater, FOBT cards, and FIT tubes and stored at −80 °C for two years [13]. The samples stored with no additive had consistently low stability ICC values for relative abundance, alpha diversity, and beta diversity measurements, whereas the other four methods all demonstrated high ICC values for relative abundance and diversity measurements when compared with immediately extracted samples. However, while these four methods showed high stability, they each demonstrated differences in phylum-level relative abundance profiles when compared to each other, demonstrating the importance of consistency in collection methods when comparing results.

Not discussed within the context of the session, additional collection and preservation methods that have been included in other methodological comparison studies include FTA cards, OMNIgene Gut, Norgen, CURNA, HEMA, Shield, and swabbed samples, as well as differences in storage temperatures [14,15,16]. All of these studies conclude that inter-subject variability in microbiome profiles is greater than variability introduced by methodological differences, but that each method does introduce some level of variability in microbiota compositional profiles.

Thus, the primary conclusions based on these findings are that the choice of collection method may depend on several factors, such as which types of analyses are planned (e.g., 16S rRNA sequencing, MGX, metabolomics), transportation temperature, and storage duration prior to and after freezing. Additionally, while several collection methods may have similar stability and concordance compared to a putative gold standard in a certain context, they each result in unique microbial and metabolite profiles. Therefore, comparison of results between studies may differ by sample collection and storage methods, highlighting the importance of transparency and clarity of methods when publishing results. The use of detailed reporting guidelines such as the “Strengthening The Organization and Reporting of Microbiome Studies” (STORMS) checklist (https://stormsmicrobiome.org) will aid in the organized and comprehensive sharing of microbiome methods [17].

## 3. Microbiome Standards

Following Dr. Vogtmann, Dr. Scott Jackson from the National Institute of Standards and Technology (NIST) presented the importance of “Standards for Microbiome Measurements” and described NIST’s creation of a human fecal microbiome standard [18] that is planned to be released in late 2023. Human fecal material is a complex mixture of hundreds, if not thousands, of different species of bacteria, viruses, human cells, animal and plant material from food, as well as a collection of small molecule metabolites. Many of these components may have clinical relevance and serve as biomarkers, but realizing these associations depends on accurate and precise multi-omic measurements. The analytical performance (e.g., accuracy, precision, sensitivity, specificity) of any method or protocol can be measured or validated by using a reference material. The principle behind the use of a microbiome reference or standard is that if you know what you’re starting with, this will help you determine and validate the analytical performance of your measurement workflow.

There are two main types of standards for human gut microbiome analysis: mock/synthetic communities and human fecal standards [18,19] (Table 2).

Mock community standards are mixtures of purified microbial cultures. They are defined microbial communities that are built from the ground up and represent “ground truth” (qualitatively and quantitatively known composition) [19]. These mock community standards can be used to assess the accuracy and precision of a given method as well as understand how methodological variables within or between labs impact results. However, mock community standards are simplistic mixtures of few microbes and are not biomimetic of real microbiome samples, which are highly complex mixtures of both microbial cells and other cellular and molecular components. Thus, mock communities have a much lower diversity and lack potential matrix effects compared to fecal microbiome samples. In contrast, human fecal standards, like the one being developed by NIST [18], are not just biomimetic of real microbiome samples, they are real microbiome samples and represent the diversity and aspects such as matrix effects present in these complex communities. However, unlike mock communities, these fecal standards are not of fully defined composition with respect to their microbial taxa. Because all microbiome metagenomic workflows introduce bias, from DNA extraction technique to bioinformatic analysis and interpretation, it is not possible to assign certified values (qualitatively or quantitatively) to the microbial taxonomic composition of the fecal material. Therefore, human fecal standards cannot be used to assess the accuracy of measurement methods. However, they can be used to assess the precision or reproducibility of measurements and the impact of methodological variables.

The criteria for manufacturing a good fecal standard are homogeneity, stability, and fitness-for-purpose. Yet, human fecal material is naturally heterogeneous, meaning that material sampled from one part of the stool will contain a different collection of microbes and molecules than material sampled from another part of the stool. Thus, the first step in the manufacturing of NIST’s human fecal microbiome standard is homogenization of pooled samples (bowel movements from multiple donors) using blending and cryomilling to ensure that each aliquot contains a comparable, representative collection of microbes and molecules. Homogeneity and stability are then assessed using a suite of measurement platforms including next generation sequencing (NGS)-based metagenomics, nuclear magnetic resonance (NMR) and mass spectrometry-based metabolomics, flow cytometry, and CFU.

In summary, using microbiome reference standards, including mock communities and standardized human fecal material, it is possible to assess the analytical performance of a microbiome measurement workflow, from sample collection to sequencing and quantification, to better understand how different methods impact results. The cost of implementing these standards ranges considerably from $100 to $1000 per unit of material depending on the material type and quantity. Labs may also prepare their own microbiome standards by preparing large batches of pooled materials (e.g., feces) that are aliquoted and frozen. Individual aliquots may then be used to show methodological consistency within their laboratory over a period of months or even years, given proper collection and storage as described in the previous section.

## 4. Microbiome Sequencing Methods

Dr. Daryl Gohl of the University of Minnesota followed to describe the current state of knowledge regarding sources of bias in the process of next-generation sequencing of the microbiome and how to reduce bias within and between laboratories [20,21]. To begin, Dr. Gohl described the Microbiome Quality Control (MBQC) project, the goal of which was to identify technical sources of variability in measurement of the human microbiome to facilitate the development of standards for reporting of methods, best practices, normalization, and meta-analysis [20]. This study highlighted significant variability in 16S rRNA gene amplicon microbiome profiles between different labs due to differences in sample handling environment, DNA extraction, homogenization, polymerase chain reaction (PCR) amplification, and sequencing [20]. However, given the MBQC study design, it was difficult to parse the effects of these separate variables on data quality and accuracy.

A systematic study by Dr. Gohl and colleagues focused on the impact of protocol variables during PCR amplification, including enzyme choice, PCR cycle number, and template concentration. These parameters have both qualitative and quantitative effects on the accuracy of profiles of mock community samples [21]. This study identified aspects of particular importance in the PCR process, such as the use of proofreading polymerases (e.g., KAPA HiFi, Q5) [21,22]. These polymerases can detect, excise, and replace mis-incorporated bases due to their 3′ to 5′ exonuclease activity [22]. The KAPA HiFi polymerase has also been engineered to have high processivity, which reduces the formation of chimeric reads [21]. Thus, proofreading polymerases can help reduce substitution error rates during PCR, as well as rescue “dropout” taxa whose templates contain primer mismatches and are thus not amplified during the PCR process [21]. In a follow-up study, Dr. Gohl and colleagues reported the primer editing activity of several proofreading polymerases and demonstrated that the extent of primer editing can be tuned through the adjustment of enzyme concentration or the strategic incorporation of phosphorothioate bonds, which block exonuclease activity in the amplification primers [22]. Thus, best practices for microbiome amplicon analysis should include the use of a proofreading, highly processive polymerase, sequencing primers should not overlap with amplification primers, template concentration should be optimized, and the number of PCR cycles should be minimized [21].

The sequencing step of microbiome analysis can be described figuratively, and sometimes literally, as a black box. Next-generation sequencers are complex liquid-handling and optical devices where the raw data, which in the case of Illumina sequencers are a collection of fluorescent images, undergo a number of transformations via proprietary software to generate the FASTQ files that are the basis for subsequent bioinformatic analyses. Thus, there are likely sources of error and bias in the DNA sequencing process that are currently unappreciated. As with assessing the accuracy of other steps of the microbiome data generation process, reference standards provide useful materials to assess the DNA sequencing process. Using synthetic standards, Dr. Gohl and colleagues conducted an investigation that demonstrated a high degree of clustering bias due to molecule length across Illumina sequencers (e.g., MiSeq, NextSeq, NovaSeq) and variability in these biases between instruments that could impact interpretation and comparison of data in certain experimental contexts (for instance, in ITS amplicon sequencing, since fungal ITS regions can vary considerably in length) [23]. To account and correct for these size biases, Dr. Gohl and colleagues are in the process of developing a novel standard based on PhiX, a sequencing quality control that is typically spiked into Illumina sequencing runs to measure error rates. This standard, which is called “PhiXtra” contains a collection of PhiX fragments with defined sizes and unique breakpoints that act as molecular barcodes for different fragment sizes. Use of this standard would allow for measurement of the size bias curve in addition to the error rate during DNA sequencing.

Identification of sources of variability and bias within the microbiome analysis workflow and understanding the effect of different methods on results is a first step to ensuring data quality. If the goal is to reproduce or compare results within or between labs, the next step is to standardize methods or develop methods that can correct for these biases. Steps that can be taken to increase intra-lab reproducibility include developing detailed standard operating protocols, a quality management system (e.g., training records, version control), and a laboratory information management system (e.g., reagent lot numbers, instruments, sample touches, timestamps) as well as implementing training and proficiency testing, routine incorporation of positive and negative controls at each step, and version-controlled software. The path towards inter-lab reproducibility is more difficult and, based on the experience of researchers in other fields, can take years to achieve [24]. Though many of the same principles of increasing intra-lab reproducibility apply, achieving inter-lab reproducibility would require substantial coordination and troubleshooting of protocols, use of consistent reagents, structured data and metadata collection, and cross-training of staff in addition to a funding source invested in these efforts [24]. As discussed later in the session, adherence to standardized protocols may not always be desirable, since this can mean failing to take advantage of improvements in technology, thereby potentially sacrificing accuracy for reproducibility. Moreover, the frequent turnover or obsolescence of sequencing platforms and library prep reagents further complicate efforts to generate reproducible data over long timescales.

Thus, understanding the effect of different library preparation and sequencing protocols on variability and bias in results, and the implementation of standardized protocols or controls to correct for these sources of variability, will allow for more accurate comparison of results between studies and verification of consistent findings. However, the benefits of standardized protocols must also be weighed against the potential compromise of decreased accuracy as a result of slower adoption of new technologies and methods.

## 5. Microbiome Quantification Methods

One of the characteristic features of microbiome sequencing methods is that they are almost always presented as compositional, meaning that the abundances of microbiota taxa are relative and not absolute [25]. However, the next speaker, Dr. Doris Vandeputte of Cornell University, described issues with compositional data, the value of absolute quantification of microbial taxa, and methods for moving from relative to absolute quantification.

Compositional data present a unique challenge of analysis and interpretation. Changes in relative abundance of taxa over time or differences in relative abundance between samples cannot provide information on microbial load or directionality of changes or differences [26]. For instance, if the relative abundance of a certain taxa is higher in one sample versus another, it cannot be determined whether the absolute abundance of that taxon is higher or if the absolute abundance of the other taxa is lower in that sample. Additionally, total microbial load cannot be determined from relative abundance. Microbial load has been shown to differ in patients with Crohn’s disease from healthy controls [26] and in soil has been associated with plant yield independent of microbial composition [27]. In Crohn’s disease, relative microbiome profiling seems to suggest that *Bacteroides* is increased. However, quantitative microbiome profiling reveals that it is a decrease in total microbial load and not a bloom of *Bacteroides* that is characteristic of individuals with Crohn’s disease [26]. Thus, microbial load is a biologically significant parameter in and of itself and quantitative microbiome profiling can provide insight into the true direction of changes in microbial taxa abundances. Quantitative microbiome profiling also improves sensitivity and reduces false discovery rate of species–species associations [26]. The integration of microbiome sequencing data with other -omics data (e.g., transcriptomics, proteomics, metabolomics) is also improved by using quantitative microbiome profiling because these other omics data are often also measured using absolute abundances.

To convert relative to absolute abundances, two extra measurements are needed: the weight per sample and the cell number per sample. Cell numbers must be standardized by the weight of the sample, taking into account the addition of any buffering solutions or other additives. To measure the cell number per sample, several methods can be used (Table 3). Parallel with sequencing, flow cytometry or quantitative PCR (qPCR) of a universal marker can be utilized. Other options used during sequencing include addition of an internal standard or a combination of an internal standard and qPCR. Providing a comparison of methods, Dr. Vandeputte explained pros and cons of these different approaches. Flow cytometry, while a commonly used method, is labor intensive and requires either fresh samples or diluted frozen samples [26]. Described as a straightforward protocol with minimal bias, considerations remain, such as formation of aggregates (i.e., cells that clump together and affect cell counts) and potential bias if cells cannot be extracted or amplified, which would cause differences in the flow cytometry results compared to sequencing results [26]. Conversely, qPCR of a universal marker, gene while easily applicable, cheap, and needing only limited resources, introduces multiple sources of bias. Sources of bias include extraction, purification, and amplification of DNA as well as bias through 16S rRNA gene copy number variation [26]. Sequencing or sequencing plus qPCR of an internal standard involve steps within the sequencing process, rather than being parallel to it as with the previously mentioned approaches. Sequencing of an internal standard that is spiked into samples prior to extraction has the benefit that no extra analysis is needed. However, a drawback of this approach is that the dose of the standard is difficult to calibrate and therefore 20–80% of sequencing efforts may be devoted to measurement of the internal standard rather than the biological sample itself [28]. To avoid this issue, another approach was developed, combining sequencing and qPCR of the internal standard [29]. In this approach, only minute amounts of internal standard are needed and therefore sequencing efforts can be concentrated on the samples [29]. A drawback is that several qPCR reactions are needed per sample to quantify both the internal standard (to deduce the DNA recovery yield) and the 16S rRNA genes (to deduce the number of 16S copies after extraction) [29].

In summary, microbial load is a clinically relevant measure of the microbiome and can be measured using several different approaches both parallel to and in combination with sequencing. In addition to its clinical relevance, quantitative microbial profiling also circumvents the issue of analyzing compositional data by providing information on the directionality of changes and magnitude of differences between samples.

## 6. Microbiome Bioinformatic Methods

Regardless of whether relative or quantitative microbial profiling methods are utilized, once data are generated from sequencing they must be processed using bioinformatics to identify taxonomic groups and other biologically informative features from sequences. This step was not directly discussed in the satellite session but remains an integral step in the process of microbiome analysis. Though methods have advanced and new methods have been developed in recent years, previously published studies comparing different bioinformatic approaches demonstrate the complexity of this process and the variability that this can introduce to the microbiome analysis process [30,31]. A more recent review by Gao and colleagues provides an overview of bioinformatic methods for gut microbiome analysis [32]. The steps involved in bioinformatics processing will differ based on the type of sequencing (e.g., 16S rRNA, 18S rRNA, internal transcribed spacer (ITS) sequencing, shotgun metagenomic sequencing, metatranscriptomic sequencing, and viromic sequencing) but generally include three core steps: (1) data preprocessing and quality control, (2) assignment and alignment, and (3) community (taxonomic and/or functional) characterization.

Many bioinformatics approaches use the same process and standards for quality control: PHRED algorithm score. For 16S rRNA sequencing, taxonomic assignment has two main approaches: operational taxonomic unit (OTU)-based analysis (e.g., UCLUST, UPARSE, CD-HIT, hcOTU, ESPRIT, ESPRIT-TREE) or amplicon sequence variant (ASV)-based analysis (e.g., DADA2, UNOISE, Deblur) [4]. OTU-based analysis uses a predefined similarity threshold (typically 97%) to cluster sequences, whereas ASV-based analysis uses denoising to deduce and remove amplification and sequencing errors, which provides a higher-resolution result [32].

Metagenomic and metatranscriptomic analyses differ in that they sequence an entire genome, rather than a single gene. There are generally two approaches to the assignment and alignment step of metagenomic and metatranscriptomic sequencing: an alignment-based approach that maps reads to known microbial reference genomes (e.g., Kyoto Encyclopedia of genes and genomes (KEGG), clusters of orthologous groups (COG)) or an assembly based approach (e.g., Meta-IDBA, IDBA-UD, MetaVelvet, and MegaHit). To obtain the most accurate results, it is often recommended to use both approaches in combination [32]. Metatranscriptomic sequencing differs in that it starts with RNA, rather than DNA, and can therefore query gene expression at a given moment and under specific conditions, rather than its potential.

Pipelines or platforms that integrate the different analytical steps into one package that are commonly used include the Galaxy server (The Huttenhower Lab; https://huttenhower.sph.harvard.edu/galaxy/, accessed on 3 May 2023) and QIIME 2 (https://qiime2.org/, accessed on 3 May 2023) [33] for 16S rRNA and metagenomic sequencing, or HUMAnN2 and MG-RAST for metatrascriptomic sequence analysis [32]. The data generated from these steps can then be analyzed using appropriate biostatistical approaches to interpret changes or differences in the microbiome as well as associations with other data types.

## 7. Microbiome Biostatistical Methods

In the following presentation, Dr. Cara Frankenfeld of the University of Puget Sound reviewed foundational biostatistical considerations and examples of statistical approaches in gut microbiome research. There are many post-processing analytical options, the choice of which is influenced by research objectives, data structure and features, as well as considerations such as study design (e.g., cross-sectional, longitudinal, etc.) and integration with other datasets (e.g., diet, metabolome, etc.).

Biostatistical methods used vary based on whether the objective of the study is to understand mechanisms by which conditions shape the microbiome (e.g., microbiome as dependent [outcome] variable), to understand mechanisms by which the microbiome influences health conditions of the host (e.g., microbiome as independent [predictor] or mediator variable), or to characterize ecological associations of microbes (e.g., network analysis).

Microbiome data are structured in matrices that relate microbiome taxa or gene abundances to samples. While simple in concept, several key features of these data make it difficult to utilize conventional statistical approaches (Table 4). These key features include compositionality [26,34,35], sparsity [34,35,36], high dimensionality [34,37], heteroscedasticity, and batch effects or other technical effects.

As has been mentioned previously, the compositional or relative nature of most microbiome data influences the analysis and interpretation of these data due to the imposition of an arbitrary total sum (100%) [26]. This has the effect that the abundances of different taxa are no longer independent of one another (i.e., relative abundance of taxon A is dependent on changes in the abundance of taxon B, even if absolute numbers of taxon A do not change) [38]. The feature of sparsity, also known as zero-inflation, means that there are a high percentage of cells in the data table that contain a zero because the specific sequence or taxon was not detected [36]. Microbiome data also typically have the feature of high dimensionality, meaning that the number of features (i.e., taxa columns) is larger than the number of observations (i.e., subject rows). This high dimensionality makes it challenging to build models to detect associations between features of the microbiome and health status because there are so few observations on which to train models. Use of diversity metrics or ordination methods such as principal coordinate analysis (PCoA) are often used to reduce this dimensionality by summarizing taxa data into a single metric or a smaller number of distance vectors that can then be visualized to inspect sample clusters and similarity or dissimilarity based on bacterial composition [35]. Heteroscedasticity is the characteristic of dependent or outcome variables (e.g., microbiota taxa) having unequal variance over a range of independent variable values (e.g., metadata) or time periods. This is common and typically dealt with by using non-parametric approaches or utilizing techniques to reduce heteroscedasticity (e.g., log transformation) before using parametric approaches. As discussed in previous sections, other important factors influencing the structure of microbiome data output and data interpretation include technical variability such as batch or protocol effects.

The choice of statistical approach is limited by the aforementioned features of microbiome data but is ultimately driven by the goals of the analysis. For example, researchers may consider a summary feature of the microbiome (e.g., diversity metric), overall composition (e.g., PERMANOVA [39], LDM), differential abundance (e.g., ANCOM-BC [38], LinDA [40]), and/or taxa relationships (e.g., network analysis).

Diversity metrics provide a summary feature of the microbiome composition. As mentioned, this is one strategy of addressing the challenge of high dimensionality through data reduction. Within microbiome analysis, alpha and beta diversity are commonly used measures of diversity. Alpha diversity is used for local diversity and reflects diversity within a single sample. Examples of alpha diversity metrics include richness (e.g., number of observed species, (e.g., ACE, Chao1), phylogenetic diversity (e.g., Faith’s phylogenetic diversity), or evenness (e.g., Shannon index, Simpson index). Choice of alpha diversity metric may depend on the structure of the data. For example, both ACE and Chao1 are non-parametric richness estimators that may be more appropriate for data displaying heteroscedasticity compared to the number of observed species. Beta diversity is a measure of the similarity or dissimilarity of communities based on the distance between pairs of samples. These distances can then be used in ordination to reduce the dimensionality of the data and visualize the distances between samples. Beta diversity metrics can be categorized as either quantitative (e.g., Bray–Curtis, Canberra, weighted UniFrac) or qualitative (e.g., binary-Jaccard, unweighted UniFrac). Quantitative metrics use feature abundance data whereas qualitative metrics only consider the presence or absence of taxa features. Use of quantitative or qualitative metrics may depend on the sparsity of data or presence of rare features within the dataset. Often, both types of metrics are used. Both alpha and beta diversity metrics produce summary measures that may then be used in further statistical analyses such as *t*-tests or linear regression to test research hypotheses.

Distance matrices produced from ordination or beta diversity measurements may also be used to compare overall community composition via methods such as permutational multivariate analysis of variance (PERMANOVA) [39]. PERMANOVA is a non-parametric, multivariate extension of ANOVA that depends on permutation distributions rather than assumptions about underlying normality of the data to determine statistical significance. Overall community composition may also be compared using the taxa abundance table via the linear decomposition model (LDM) [41], which can be applied to both relative abundance data or presence-absence data when associated taxa are relatively rare.

Differential abundance analysis aims to identify differences or changes in the abundance of individual taxa between samples or over time. As mentioned previously, this can be complicated by the compositional or relative nature of most microbiome data. One of the strategies to address this issue is through robust normalization. Once normalized, the resulting values can then be used in statistical procedures such as regression or correlation. One normalization strategy involves calculating some normalizing factor that is robust to a small number of differential taxa, then dividing by this normalizing factor. This will bring the abundance of non-differential taxa to the same scale and retain differences between differential taxa. Procedures that incorporate this normalizing factor approach include edgeR [42] and MetagenomeSeq2 [43]. Another common approach is the center log ratio (CLR) transformation in which the counts are divided by their geometric means before taking the logarithm of these values. This approach is used in ANCOM and ANCOM-BC [38]. A new method, LinDA [40], also allows for correlated or clustered data such as would arise from paired or repeated measures, which is not available in ANCOM-BC. However, LinDA does have a false discovery rate inflation that may not be appropriate for datasets with small features sizes such as phylum level abundance data. Thus, it is important to consider the structure of the dataset when deciding which method to use for differential abundance testing. Recent publications have compared various differential abundance methods [44,45,46,47].

The microbiota is a complex ecosystem in which individual taxa interact, positively and negatively, to form an ecological network of co-occurring microbes. Thus, while comparison of individual taxa can be informative, network analysis can provide insight into these microbial interaction patterns [48]. There are four common categories of network analysis models: correlation and regression based methods, local similarity analysis, probabilistic graph models, and matrix factorization techniques [48]. Correlation and regression based methods utilize additional steps such as normalization to account for the compositionality of microbiome data as well as bootstrapping and penalty terms (e.g., lasso penalty to drive the coefficients of taxa with negligible contributions to zero) to account for the high dimensionality and sparsity of microbiome data [48]. Local similarity analysis detects changes between time series measurements of bacteria to identify associations and study dynamic changes in microbial communities [48]. Probabilistic graph models map microbial interactions and, contrary to correlation- and regression-based methods, differentiate between direct and indirect associations [48]. Matrix factorization techniques infer low dimensional structure (e.g., principal components or coordinates) from high dimensional -omics data to enable visualization and inference of relationships between the microbiome and health outcomes. The tools most useful for a specific analysis depend on characteristics of the data such as compositionality, sparsity, dynamic/time series, indirect and nonlinear interactions, differential analysis, and/or multiple networks [48].

Complex extensions to microbiome analysis such as longitudinal analysis and integration with other complex data pose additional challenges. A review by Kodicara et al. [49] discusses the challenges that microbiome data pose for longitudinal analysis and provides a comparison of methods for longitudinal analysis using simulated data. Integration of other data sets such as -omics or dietary data presents a challenge given the baseline complexity of microbiome data in combination with the complexity of dietary or other -omics data. A review by Choi et al. [50] demonstrates the complexity of integrating microbiome and dietary data, also illustrating the parallels in analysis pipeline steps of data collection, data preparation, data processing, data structure, and output between microbiome and dietary data such as compositionality and sparsity. Choi and colleagues also highlight an approach called Procrustes analysis that can be used to compare microbiome ordination with dietary ordination to determine if there is agreement between the two sets of data, though this method is limited by the inability to adjust for covariates [50]. A review by Bhosle and colleagues [51] delves into the use of machine learning approaches such as MimeNet, NPLinker, and MelonnPan to find or predict microbe–metabolite interactions. In a more general approach to integrating -omics datasets, Ghazi and colleagues [52] describe methods of identifying associated features between different -omics datasets including parametric methods (e.g., partial least squares [PLS], canonical correlation analysis [CCA], sparse principal component analysis [SPCA], and SPARSE-CCA), non-parametric methods (distance correlation [dCor] and Chatterjee rank correlation [XICOR]), and a novel method of linking high-dimensional -omics datasets called hierarchical all-against-all association testing (HAllA).

Ultimately, each statistical method has a designated purpose and limitations that must be considered along with the research objectives and data structure to determine which methods are appropriate for analysis. Use of appropriate statistical methods to analyze individual datasets as well as compare or link datasets provides a powerful avenue for understanding mechanistic pathways and establishing cause and effect between diet, microbiome, and health outcomes.

## 8. Diet-Microbiome-Health Interactions: Considerations for Research

**Figure 1:**
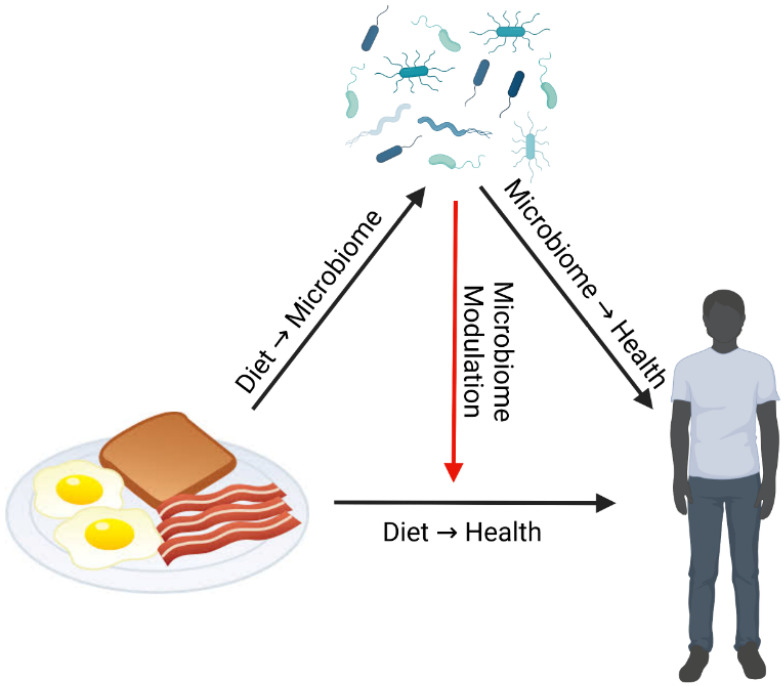
Diet–Microbiome–Health Interactions. Direct effect arrows are black, indirect effect arrow is red. Diet has direct effects on both health and the gut microbiome. The gut microbiome also has a direct effect on health as a result of changes in the community composition and/or function due to diet. However, the gut microbiome can also modulate (indirect effect) the effects of diet on health independent of diet-related changes in composition or function.

Dr. Curtis Huttenhower reviewed how microbiome data can be utilized within diet-microbiome studies. Any perturbation such as diet can directly affect both health and the gut microbiome (Figure 1). Gut microbial composition and function can respond directly to diet, and in turn gut microbes can directly affect health [53]. However, the gut microbiome can also affect health by modulating the effects of diet, not necessarily with a corresponding change in the composition of the microbiome itself [5,54]. This last interaction has garnered particular interest in the microbiome as a component of personalized nutrition [54,55,56]. For instance, the gut microbiome has been found to modulate individual glycemic response after standardized meals [5], cardiometabolic risk in response to a Mediterranean diet [54], and inflammation in response to dietary fiber [57].

In fact, while diet does directly affect gut microbiome composition and function, the effect sizes can in many settings be smaller than expected, even if they are statistically significant [54]. Diet can be a dominant influence on gut microbiome composition and function in certain contexts and populations such as in infants [58], hunter-gatherer populations [59], non-human primates [60], and in lab mice with highly controlled diets [61]. However, in most healthy human adult populations eating a westernized diet (i.e., highly varied and not subject to seasonal cycling of food availability) [62,63,64,65,66], dietary differences seem to have only modest effects on the gut microbiome. Thus, in some contexts it may be modulation of dietary effects by the baseline gut microbiome that matter more than direct effects of diet on the gut microbiome in terms of health outcomes. For example, in a study by Wang et al., while the Mediterranean diet showed modest effects on gut microbiome composition and function, there was a strong interaction between individuals’ baseline abundance of *Prevotella copri* and the effect of a Mediterranean diet on cardiometabolic health [54]. Individuals who had higher relative abundance of *P. copri* were cardiometabolically protected regardless of Mediterranean diet adherence, while individuals with lower abundance were more dependent on diet adherence for cardiometabolic health [54]. Similarly, *P. copri* abundance has demonstrated the same interaction with fiber intake and plasma C-reactive protein concentration (i.e., inflammation) [57].

In summary, diet can have direct effects on the gut microbiome and health outcomes. However, in many healthy adult human populations, modulation of dietary effects on health by the gut microbiome may play a bigger role in various health phenotypes. This highlights the importance of personalized nutrition approaches that focus on or account for baseline gut microbiome composition and function.

## 9. Discussion

This ASN satellite session reviewed the current state of knowledge related to methods used for microbiome analysis: collection, storage, sequencing, bioinformatic processing, and biostatistical analysis. In all steps of this analytic process, the importance of methodological synchronicity or use of standards for comparability of results as well as the consideration of research objectives to determine appropriate methods were highlighted as key factors in microbiome research.

Collection and storage methods are the first step in microbiome research and are the foundation for ensuring quality results. Choice of method depends on several factors including what subsequent analyses are planned (e.g., 16S rRNA sequencing, MGX, metabolomics). Thus, the research objectives or subsequent planned analyses factor into the choice of collection method. In the case of multi-omic analyses, best storage and collection practices for each individual analysis pipeline remain the same and individual aliquots should be treated accordingly. Multi-omic data may then be integrated during the biostatistical analysis step as discussed. Additionally, while several collection methods may have similar stability and accuracy compared to a putative gold standard in a certain context, they each result in unique microbial and metabolite profiles. Therefore, comparison of results must consider collection methodology, and standardization of collection and storage should be sought among studies with concordant research objectives and subsequent analytic steps. Use of standards (e.g., mock community standard, defined human fecal standard) during collection and sequencing can help identify differences between methods and help refine and optimize technological approaches.

In addition to the use of standards during the sequencing process, use of other tools can also reduce bias generated from the sample handling environment, DNA extraction, homogenization, and PCR amplification that are part of the sequencing process. For example, the use of proofreading polymerases during PCR amplification can reduce errors and bias that could impact downstream sequencing results. While the use of standards and other tools such as proofreading polymerases can reduce bias, standardization of sequencing protocols is important to achieve inter-study comparability of results and to identify and validate associations and trends within the microbiome. Additionally, quantification of absolute microbe abundances, rather than the relative abundance that arises from the standard sequencing process, may also be a step forward to identify associations within the microbiome and enable improved interpretation and comparability of results.

The bioinformatic and biostatistical approaches that are employed downstream of sequencing form the capstone of the microbiome analytic process, define the ultimate interpretation, and inform the narrative that accompanies the data. Both bioinformatic and biostatistical steps require consideration of the characteristics of the data input and the desired data output or research objectives as these considerations drive the choice of parameters and methods. Specific bioinformatic parameter recommendations (e.g., trimming length, truncating length, or sampling depth) were not covered during the satellite session. However, choices about bioinformatic parameters should be made based on the quality of the sequences in the dataset as well as the expected abundance of the taxa of interest (i.e., is the research questions focused on rare or abundant taxa). As noted, researchers should select their method of microbiome statistical analysis to align with their research question. For example, a question about identifying specific differences in microbiome species would be analyzed differently than a question about understanding global changes in the microbiome profile. Once the main method of analysis is selected to align with the research question, researchers should consider features of their specific dataset, such as magnitudes of sparsity and dimensionality. Microbiome analysis is a rapidly evolving field and microbiome scientists are developing and releasing new and adapted methodologies. Particular methods or extensions to methods may be available for a specific type of analysis that are better suited to address higher sparsity or dimensionality. For example, in a recent publication, Rahman et al. [67] introduced a flexible differential abundance method that utilizes Bayesian approaches to account for high sparsity, high dimensionality, and compositionality. Researchers are encouraged to evaluate longer-standing statistical tools and recent additions to the statistical toolbox, and validation studies of such tools, to identify what extensions to analytic methodologies would align best with the features of their specific datasets.

In summary, choice of methods is dependent on research objectives and often limited by available resources. Continued development, validation, and standardization of methods can help move microbiome research forward by increasing accuracy and precision of results. Clear description of methods by researchers in the dissemination of their results, will enable greater inter-study comparability and validation of findings.

## Figures and Tables

**Table 1:** Microbiome sample collection methods.

Method	Pros	Cons
No additive	No extra additives needed	Least stable (low ICC)
70% ethanol		Low stability (low ICC)
95% ethanol	High stability (high ICC) with 16S rRNA gene amplicon sequencing and metabolomic analysis Most metabolites detected with metabolomic analysis	Lower stability (low ICC) with MGX
RNAlater	High stability (high ICC) with 16S rRNA gene amplicon sequencing and MGX	Cannot be used in metabolomic analysis due to high sodium sulfate content
FOBT cards	High stability (high ICC) with 16S rRNA gene amplicon sequencing, MGX, and metabolomic analysis	
FIT tubes	High stability (high ICC) with 16S rRNA gene amplicon sequencing and MGX	Lower stability (low ICC) with metabolomic analysis

Abbreviations: fecal immunochemical test (FIT), fecal occult blood test (FOBT), intraclass correlation coefficient (ICC), shotgun metagenomic sequencing (MGX).

**Table 2:** Microbiome standards methods.

Method	Pros	Cons
Mock/synthetic communities	Represent “ground truth” (qualitatively and quantitatively known composition) Can be used to assess accuracy and precision/reproducibility of methods	Simplistic mixtures of few microbes lacking diversity and matrix effects (not biomimetic of human microbiomes)
Human fecal standards	Composed of real microbiome samples so are of representative diversity and matrix effects Can be used to assess precision/reproducibility of methods	Composition not fully qualitatively or quantitatively defined so cannot be used to assess accuracy of methods

**Table 3:** Quantitative microbiome profiling methods.

Method	Relative to Sequencing	Pros	Cons
Flow cytometry of bacterial cells	Parallel	Straightforward protocol with minimal bias	Labor intensive Requires fresh samples Formation of aggregates Potential bias if cells cannot be extracted/amplified
qPCR of universal marker gene	Parallel	Easily applicable Limited resources needed	Bias through extraction, purification, and amplification of DNA Bias through 16S rRNA gene copy number variation Bias through community replication rate
Sequencing internal standard	During	No extra analysis needed	20–80% of sequencing efforts devoted to measuring internal standard Dose of internal standard difficult to calibrate
qPCR of internal standard	During	Small amount of internal standard needed Sequencing efforts concentrated on sample	Several qPCR reactions needed per sample

Abbreviations: quantitative polymerase chain reaction (qPCR).

**Table 4:** Features of microbiome data.

Microbiome Data Feature	Definition
Compositionality	Data are relative (percentage or proportion). Abundances of taxa are no longer independent of one another.
Sparsity	Also known as zero-inflation. A high percentage of cells in the data contain a zero because the specific sequence or taxon was not detected.
High dimensionality	The number of features (i.e., taxa columns) is larger than the number of observations (i.e., subject rows).
Heteroscedasticity	Dependent or outcome variables (e.g., microbiota taxa) display unequal variance over a range of independent variable values (e.g., metadata) or time periods

## Data Availability

Not applicable.
